# Notice of Retraction and Replacement. Wang L, et al. Opportunistic Screening With Low-Dose Computed Tomography and Lung Cancer Mortality in China. *JAMA Netw Open.* 2023;6(12):e2347176

**DOI:** 10.1001/jamanetworkopen.2024.38532

**Published:** 2024-09-30

**Authors:** Fuzhong Xue

**Affiliations:** 1 Department of Biostatistics, School of Public Health, Cheeloo College of Medicine, Shandong University, Jinan, China; 2 Healthcare Big Data Research Institute, School of Public Health, Cheeloo College of Medicine, Shandong University, Jinan, China; 3 Qilu Hospital, Cheeloo College of Medicine, Shandong University, Jinan, China

**To the Editor** On behalf of my coauthors, I write to explain errors in the Original Investigation, “Opportunistic Screening With Low-Dose Computed Tomography and Lung Cancer Mortality in China,”^1^ published in *JAMA Network Open* on December 12, 2023. In this study, we aimed to evaluate whether opportunistic low-dose computed tomography (LDCT) screening was associated with improved prognosis among 5234 adults with lung cancer in China. Our cohort included patients diagnosed with lung cancer who were classified into screened and nonscreened groups on the basis of whether or not their lung cancer diagnosis occurred through opportunistic screening. We reported that opportunistic screening with LDCT was significantly associated with a 49% lower risk of lung cancer death and 46% lower risk of all-cause death.

After publication, readers posted comments with concerns about and symptomatic vs opportunistic screening^2^ and potential lead time bias and length bias.^3^ Following discussion with the *JAMA Network Open* editors, we conducted new analyses to address these concerns. To address the concern raised by Dr Hammer^2^ about symptomatic vs opportunistic screening, we included the tumor characteristics into the propensity score model. To address concerns noted by Drs Stumpf and Rustagi’s concerns^3^ about lead time and length biases, we used methods proposed by Duffy et al.^4^ Accordingly, to correct for lead time bias, we subtracted the expected lead time from the observed survival time for each patient in the screened group. We found large variation in reported lead times in the literature^5,6^; therefore, we focused on an intermediate lead time of 210 days and comprehensively studied 150, 180, 240, and 270 days for sensitivity analysis. To correct for length bias, we first followed Duffy et al’s method to calculate the unadjusted for confounding hazard ratio (HR) with and without correcting for length bias, which depends on the anticipated lead time bias. These results were used to compute a length bias correction factor, which was then applied to the HR estimates obtained after correction for confounding and lead time biases. By using this combined, ad hoc approach, all the 3 of biases were addressed in the final HR estimates. With these new analyses, we continue to find lower risk of lung cancer death and of all-cause death. However, we now find that opportunistic screening with LDCT was associated with a 34% lower risk of lung cancer death (HR, 0.66; 95% CI, 0.54-0.80) and 28% lower risk of all-cause death (HR, 0.72; 95% CI, 0.60-0.86).

We thank the readers for sharing their concerns and the journal editors for allowing us to retract and replace the original article with this corrected version. All authors agree with this. We hope this explanation is helpful to readers. Corrections to the original article have been made to the Abstract, main text, Figure 3, and Supplement 1, and we have added a new Table 2 to show the findings of the new analysis after adjusting for confounding, lead time bias, and length bias.^1^ New Supplements have been added to show the original article with errors highlighted and the corrected article with corrections highlighted.
